# Preparation and Properties of Various Magnetic Nanoparticles

**DOI:** 10.3390/s90402352

**Published:** 2009-03-30

**Authors:** Jana Drbohlavova, Radim Hrdy, Vojtech Adam, Rene Kizek, Oldrich Schneeweiss, Jaromir Hubalek

**Affiliations:** 1 Department of Microelectronics, Faculty of Electrical Engineering and Communication, Brno University of Technology, Údolní 53, 602 00 Brno, Czech Republic; E-Mails: hrdyr@email.cz (R.H.); hubalek@feec.vutbr.cz (J.H.); 2 Department of Chemistry and Biochemistry, Faculty of Agronomy, Mendel University of Agriculture and Forestry, Zemědělská 1, 613 00 Brno, Czech Republic; E-Mails: ilabo@seznam.cz (V.A.); kizek@sci.muni.cz (R.K.); 3 Institute of Physics of Materials, Academy of Sciences of the Czech Republic, Zizkova 22, 616 62 Brno, Czech Republic; E-Mail: schneew@ipm.cz (O.S.)

**Keywords:** Magnetic nanoparticles, iron oxide, gadolinium nanoparticles, silica coating

## Abstract

The fabrications of iron oxides nanoparticles using co-precipitation and gadolinium nanoparticles using water in oil microemulsion method are reported in this paper. Results of detailed phase analysis by XRD and Mössbauer spectroscopy are discussed. XRD analysis revealed that the crystallite size (mean coherence length) of iron oxides (mainly γ-Fe_2_O_3_) in the Fe_2_O_3_ sample was 30 nm, while in Fe_2_O_3_/SiO_2_ where the ε-Fe_2_O_3_ phase dominated it was only 14 nm. Gd/SiO_2_ nanoparticles were found to be completely amorphous, according to XRD. The samples showed various shapes of hysteresis loops and different coercivities. Differences in the saturation magnetization (MS) correspond to the chemical and phase composition of the sample materials. However, we observed that MS was not reached in the case of Fe_2_O_3_/SiO_2_, while for Gd/SiO_2_ sample the MS value was extremely low. Therefore we conclude that only unmodified Fe_2_O_3_ nanoparticles are suitable for intended biosensing application *in vitro* (e.g. detection of viral nucleic acids) and the phase purification of this sample for this purpose is not necessary.

## 1. Introduction

Magnetic nanosized particles have already been known for over 50 years, but research into their potential use in medicine and pharmaceutics is now the hot topic in this domain [1,2]. The unique combination of high magnetization and paramagnetic behaviour opens these materials to a very wide range of applications. Particularly, the possibilities of nanoparticle modification by biologically active compounds to use them in controlled drug delivery systems, as agents in magnetic resonance imaging and for magnetic-induced tumor treatment via hyperthermia are very interesting [3]. Iron oxide based-nanoparticles belong to the most widely used materials in this field, although they have worse magnetic properties, lower saturation magnetization, and lower specific loss of power than Fe and Co nanoparticles which have just started to gain attention for biomedical purposes, too [4]. However, iron oxides have several advantages over Fe and Co nanoparticles, e.g., better oxidative stability, compatibility in nonaqueous systems, and nontoxicity. Among the four well-known crystalline polymorphs of iron(III) oxide (α-Fe_2_O_3_ as hematite, β-Fe_2_O_3_, γ-Fe_2_O_3_ as maghemite and ε-Fe_2_O_3_), maghemite has gained the greatest interest in above mentioned applications [5]. Moreover, magnetite Fe_3_O_4_ is also very promising candidate as it is biocompatible and biodegradable [6,7].

Several methods are generally employed for iron oxide nanoparticle preparation, including co-precipitation [8], which is preferred due to its simplicity. On the other hand, thermal decomposition [9] seems to give the best control of nanoparticles size and morphology. The resulting physico-chemical properties of nanosized magnetic product obviously depend strongly on the fabrication conditions, especially on material origin, concentration and pH of solution as well as on the mode of thermal treatment used (annealing temperature, atmosphere and rate of heating/cooling). It was found that ferromagnetic low temperature phase γ-Fe_2_O_3_ can be easily transformed into the antiferromagnetic more stable phase α-Fe_2_O_3_ when the temperature exceeds 500 °C [10]. Thus it is extremely important to optimize the preparation procedure in order to prevent formation of undesired product(s). The particle size also plays a crucial role. Typical particle sizes for the ferro- to superparamagnetic phase transformation are between 10 and 20 nm for oxides and 1–3 nm for metals [11]. Morales *et al*. observed that the use of polymers in the material synthesis limits the particle size [12]. Ultrasmall magnetic iron oxide nanoparticles (<5 nm) with very uniform size distribution can be also synthesized using the water-in-oil microemulsion method [13].

Recently, more sophisticated Fe_2_O_3_ nanoparticles were fabricated where the magnetic core was covered by an amorphous silica shell [14]. The frequently used raw materials for Fe_2_O_3_/SiO_2_ preparation are iron salts (chlorides [15], nitrates [16], sulphates [17] etc.) and various silicates (water glass, sodium metasilicate). In the case of the sol-gel preparation technique employing TEOS (tetraethyl orthosilicate) as silica source, it was discovered that the particle size was independent of the silica host matrix porosity, but strongly dependent on the amount of solvent trapped inside the gels [18]. The silica shells could be further modified for better conjugation with various biological molecules such as antibodies, proteins, targeting ligands etc. [19]. From the tumor diseases treatment point of view, –NH_2_ and –SH groups are particularly interesting, because they can provide easy coupling of magnetic nanoparticles with various biologically important molecules such as the promising tumor disease marker called metallothionein [20]. Streptavidin is another important material which can be immobilized on magnetic nanoparticles in order to use them for biosensing purposes [21]. Streptavidin is known for its special affinity towards the vitamin biotin and hence it is suitable for detection of diverse biomolecules in immunoassays, e.g. detection of viral nucleic acids *in vitro*.

Moreover, it was found that the combination of SiO_2_ core and protecting coating was useful for designing paramagnetic gadolinium nanoparticles for multimodal contrast agent with optical and magnetic properties [22]. However, the synthesis of such products is often time-consuming, so there is a demand for using rather simpler ways of fabricating magnetic nanoparticles. This paper is aimed at the study of basic magnetic properties of silica coated and non-coated iron oxide prepared with the help of a simple co-precipitation method compared to gadolinium nanoparticles in silica matrix fabricated using a water in oil microemulsion system.

## 2. Experimental Section

To prepare Fe_2_O_3_/SiO_2_ nanoparticles, we employed an easy co-precipitation method reported previously by Ichiyanagi *et al*. [23]. Briefly, a 0.05 M aqueous solution of FeCl_2_·4H_2_O (Fluka) was mixed with 0.02 M aqueous solution of Na_2_SiO_3_·9H_2_O (Reachim) at pH 7. The formed black colored precipitate was washed with distilled water, dried at 80 °C for 15 min and finally air-annealed for 4 hours at 800 °C in an oven.

The following process was applied for the fabrication unmodified Fe_2_O_3_ magnetic particles: 0.05 M aqueous solution of FeCl_2_·4H_2_O was mixed with a solution containing 1g/L of K_2_CO_3_ (Penta) under constant stirring up to pH 7, which resulted in the formation of a black precipitate. After washing and separation, the precipitate was dried at 80 °C for 15 min, then finely crushed in an agate mortar and finally treated thermally at 200 °C in air in an oven for 4 hours.

The process of Gd/SiO_2_ nanoparticles synthesis published recently by Santra *et al*. [24] was slightly modified in our work, i.e. without final amine-functionalization and using similar reagents: *N*-[3-(trimethoxysilyl)propyl] ethylenediamine for capturing of paramagnetic Gd^3+^ ions and 3-(trihydroxy-silyl)propyl methylphosphonate monosodium salt solution to produce highly water dispersible nanoparticles. All reagents were purchased from Aldrich, except NH_4_OH (Fluka).

Approximate particle size of samples was determined using Scanning Electron Microscopy (SEM, model FEI Quantum 200). For SEM analysis, samples were placed on conductive copper sticking tape. The structure of the samples was checked by X-ray diffraction (XRD) using X’PERT diffractometer from PANalytical and CoKα radiation with qualitative analysis carried out by HighScore software and the JCPDS PDF-2 database. For a quantitative analysis of the XRD patterns, we took HighScore plus with Rietveld structural models based on the ICSD database. ^57^Fe Mössbauer spectra used for phase analysis were measured using ^57^Co/Rh source in standard transmission at room temperature and in a cryostat down to 28 K. Spectrum calibrations were done using α-Fe standard. The computer processing of the spectra yielded intensities *I* of the components (atomic fraction of Fe atoms), their hyperfine inductions *B*_hf_, isomer shifts *δ*, quadrupole splittings Δ*E*_Q_, and quadrupole shifts *ε*_Q_. The magnetic measurements were carried out using vibrating sample magnetometer at room temperature in an external magnetic field up to 1 T.

## 3. Results and Discussion

**Figure 1.**
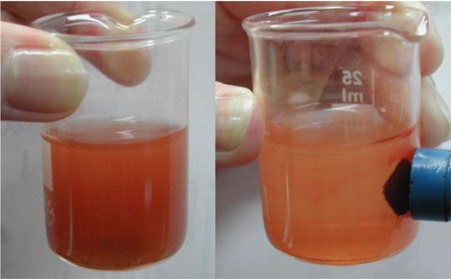
Magnetic properties illustration of Fe_2_O_3_ nanoparticles dispersed in water.

The obtained Fe_2_O_3_/SiO_2_ and Fe_2_O_3_ powders were brownish-black and reddish-brown, respectively, while Gd/SiO_2_ nanoparticles were orange due to their doping by fluorescent dye, tris(2,2′-bipyridyl) dichlororuthenium(II) hexahydrate. Only Fe_2_O_3_ nanoparticles revealed the magnetic properties in water suspension when external magnetic field was applied (Figure 1.).

**Figure 2.**
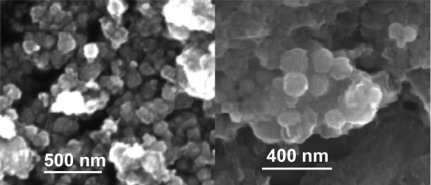
SEM image of Fe_2_O_3_ (left) and Gd^III^/SiO_2_ (right).

SEM analysis demonstrated that the nanoparticles of all prepared samples have particle sizes below 100 nm (Figure 2.). The particles were generally spherical in the shape. However it can be supposed that the size distribution for all samples is rather wide.

**Figure 3.**
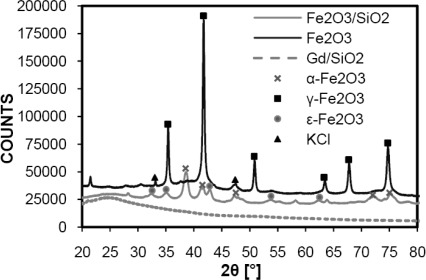
XRD patterns of Fe_2_O_3_/SiO_2_, Fe_2_O_3_ and Gd^III^/SiO_2_.

According to XRD measurement (XRD patterns of all samples are shown in Figure 3.), Gd^III^/SiO_2_ nanoparticles were found to be completely amorphous. The orthorhombic ε-Fe_2_O_3_ was observed as majority phase (49 %) together with hematite (24 %) in the case of Fe_2_O_3_/SiO_2_ annealed at 800 °C for 4 hours, which is different from the results reported by Ichiyanagi *et al*. [25]. The ε-Fe_2_O_3_ phase is considered to be an intermediate between maghemite and hematite and it is magnetically very interesting for its high coercive force [26]. The value of mean coherence length corresponding to crystallite size estimated using Rietveld analysis was about 14 nm for both α- and ε-Fe_2_O_3_ phases. It can be assumed that there are also very small maghemite or magnetite particles but these were not detected in XRD spectra probably due to “hiding” of their peaks in the amorphous one belonging to SiO_2_ (27 %).

**Figure 4.**
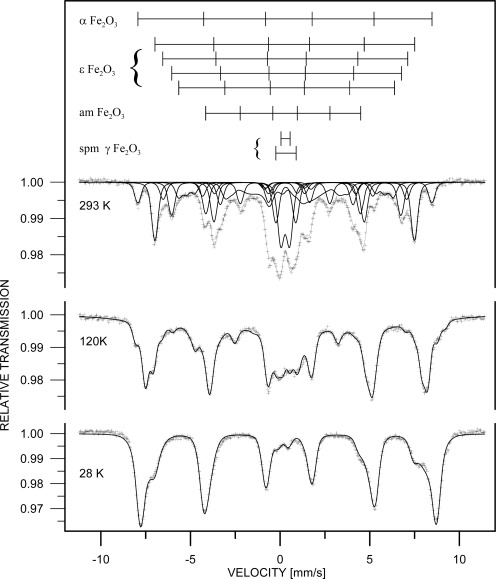
Mössbauer spectrum of Fe_2_O_3_/SiO_2_ sample depending on measurement temperature changes.

The phase analysis carried out from the Mössbauer spectrum (Figure 4) confirmed ε-Fe_2_O_3_ as the dominating iron containing phase (51 %) represented by four sextets with following parameters (see Table 1.). The ε-Fe_2_O_3_ phase was completed by hematite (8 %), superparamagnetic maghemite (19 %, represented by two doublets) and amorphous phase (22 %, represented by a broad sextet).

The superparamagnetic behaviour of the maghemite is documented by the measurement of the Mössbauer spectra at various temperatures (shown in Figure 4.). The doublets ascribed to the maghemite splitted continuously to sextets when the sample temperature decreased below the blocking temperature. The broad interval of the blocking temperature indicates broad size distribution of the maghemite nanoparticles. The amorphous phase represents probably surfaces, interfacial regions, fine particles slightly below blocking temperature and particles with perturbed oxygen stoichiometry of the magnetic phases mentioned above. Generally, all values of relative intensities and hyperfine parameters agree with the data reported in literature, e.g. [10,27,28].

**Figure 5.**
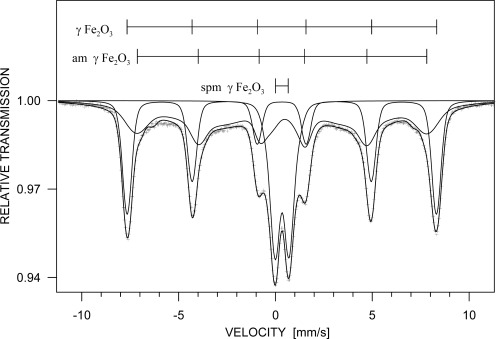
Mössbauer spectrum of Fe_2_O_3_ sample.

The prepared unmodified Fe_2_O_3_ powder consisted of 60 % iron oxide, 30 % of sylvite (KCl) and small portion of amorphous phase. The mean coherence length of iron oxide phase was about 30 nm. Since X-ray powder diffraction cannot distinguish between maghemite and magnetite nanoparticles, Mössbauer spectroscopy analysis was performed to distinguish these crystallographic modifications (Figure 5). The results showed the maghemite phase being dominant (36 %), as represented with a sextet of *B*_hf_ = 49.6±0.1 T, *δ* = 0.32±0.01 mm/s, *ε*_Q_ = 0.01±0.01 mm/s). Besides that a small amount of magnetite Fe_3_O_4_ (6 %, two sextets *B*_hf_ = 48.1±0.1 T, *δ* = 0.32±0.02 mm/s, *ε*_Q_ = 0.06±0.03 mm/s and *B*_hf_ = 45.1±0.1 T, *δ* = 0.67±0.02 mm/s, *ε*_Q_= −0.07±0.03 mm/s) and some amorphous phase which is probably of the same origin as in the case of Fe_2_O_3_/SiO_2_ were detected. Generally it is very difficult to distinguish between a true amorphous Fe_2_O_3_ phase and nanocrystalline polymorphs exhibiting very small particle size [29]. Thus for their more precise identification, a measurement in external magnetic field should be done. Moreover, a superparamagnetic or paramagnetic component was detected in this sample.

**Figure 6.**
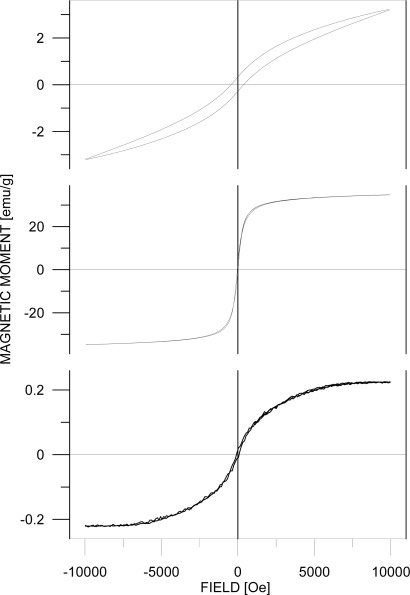
Magnetization curves of Fe_2_O_3_/SiO_2_ (top), Fe_2_O_3_ (center) and Gd^III^/SiO_2_ (bottom).

Magnetic hysteresis loops taken at room temperature are shown in Figure 6. The hysteresis loop (HL) of Fe_2_O_3_/SiO_2_ nanoparticles documents that we were not able to saturate the sample in the field of 10 kOe. Therefore we can only report the parameters of the minor HL at the field of 10 kOe: moment of 3 emu/g and corresponding coercive field of 400±20 Oe. It indicates that the phase did not reach the particle size where its shape and magnetocrystalline anisotropies cause high coercivity. The moment value could be recalculated to the pure ε-Fe_2_O_3_ from the phase composition given by XRD and Mössbauer phase analysis. In order to reach saturation on the HL, a strong external field is necessary [30]. On the other hand, such a strong external field would influence the mutual interactions of the ε-Fe_2_O_3_ and the (super)paramagnetic phase (at room temperature) which is present in the sample according to the Mössbauer spectrum measurement.

The HL shape of the Fe_2_O_3_ nanoparticles corresponds well to an assembly of individual magnetic particles with mutual dipolar interaction. The value of magnetic moment at 10 kOe is about 35 emu/g. Taking into account the results of phase analysis, i. e. the contents of sylvite (∼30% according to XRD) and (super)paramagnetic phase (∼25% from Mössbauer spectrum), we can roughly estimate the moment of γ-Fe_2_O_3_ nanoparticles to be 66 emu/g. This is a comparable value to that reported in [31] and to the one inside the range of values 60–80 emu/g published in [32]. The coercive field derived from the HL is about 30±5 Oe and it roughly agrees with data described in [33].

The HL parameters of Gd^III^/SiO_2_ sample are the moment of 0.23 emu/g at 10 kOe and coercive field of 70±5 Oe. Estimation of the magnetic moment to a pure Gd phase from the expected phase composition given by chemical composition would increase the moment value approximately two times. This is still substantially lower value than the one reported for bulk Gd (139 emu/g) [34]. It should be mentioned that Curie temperature for pure Gd is about 289 K. A comparison to non-protected Gd nanoparticles or thin films (e.g. to those described in [35]) would not be correct because of probably important differences in Gd sample oxidations during the most frequently applied preparation techniques and/or handling of the samples in ambient atmosphere.

## 4. Conclusions

In this article we report the synthesis of iron oxides and gadolinium nanoparticles using co-precipitation and water in oil microemulsion methods, respectively. The XRD phase analysis showed interesting differences in development of crystallinity and mean coherent lengths. While in Fe bearing materials crystalline phase peaks were detected, the Gd based sample only showed an amorphous diffraction. The ε-Fe_2_O_3_ was found to be the dominating crystalline phase in the Fe_2_O_3_/SiO_2_ sample. This result was confirmed by Mössbauer phase analysis. Maghemite dominated in the unmodified Fe_2_O_3_ powder sample. The magnetization curves corresponded to assemblies of individual magnetic particles with mutual dipolar interaction separated by another nonmagnetic phase, e.g. SiO_2_ and KCl. The samples exhibited clear differences in saturation magnetization which can be ascribed mainly to the different chemical composition and magnetic moments of Fe and Gd and their phases. Since ε-Fe_2_O_3_ phase did not reach a particle size where its shape and magnetocrystalline anisotropies cause high coercivity, one of the objectives in future work will be to increase the coercive force, as well as the amount of this phase by prolongation of sample annealing time.

We found that only the Fe_2_O_3_ sample is suitable for practical medical applications due to its sufficiently high value of saturation magnetization and attraction to magnet ability. Despite the fact that this material does not consist of one phase, it can be used for biosensing purposes *in vitro*, for example for detection of viral nucleic acids (influenza, jaundice) with the help of streptavidin-conjugated magnetic nanoparticles. We expect the surface of this sample can be modified further by specific sequences of nucleic acids complementary to viral DNA in order to detect the serious diseases such as cancer, HIV, bird flu etc. We also propose these nanoparticles can be coated by chicken antibodies against metallothionein, which can specifically interact with the target molecule.

## Figures and Tables

**Table 1.** Hyperfine parameters deduced from Mössbauer spectrum of Fe_2_O_3_/SiO_2_ measured at 293 K. (c – phase content as atomic fraction of iron atoms, *I* – fraction of the component, *B*_hf_ – hyperfine induction, *δ* – isomer shift, *ε*_Q_ – quadrupole splitting, Δw – width of the hyperfine field distribution; spm. – superparamagnetic, am. – amorphous).

**Phase**	**c [%]**	**Component**	***I***	***B*_hf_ [T]**	***δ* [mm/s]**	***ε*_Q_ [mm/s]**	**Δw [T]**

ε-Fe_2_O_3_	51±1	1st sextet	0.42±0.01	45.0±0.1	0.37±0.01	−0.25±0.01	-
		2nd sextet	0.12±0.01	42.2±0.2	0.28±0.03	−0.02±0.01	-
		3rd sextet	0.24±0.01	39.7±0.2	0.35±0.01	−0.03±0.01	-
		4th sextet	0.22±0.01	26.8±0.1	0.22±0.01	−0.11±0.01	-

α-Fe_2_O_3_	8±1	sextet	1.00	51.3±0.1	0.34±0.01	−0.17±0.02	-

γ-Fe_2_O_3_ spm.	19±1	1st doublet	0.60±0.01	-	0.28±0.01	0.97±0.03	-
		2nd doublet	0.40±0.01	-	0.32±0.01	2.21±0.04	-

am.	22±1	sextet	1.00	11.9±0.3	0.35±0.04	−0.20±0.06	7.3±0.8
